# Changes in miRNA in the lung and whole blood after whole thorax irradiation in rats

**DOI:** 10.1038/srep44132

**Published:** 2017-03-17

**Authors:** Feng Gao, Pengyuan Liu, Jayashree Narayanan, Meiying Yang, Brian L. Fish, Yong Liu, Mingyu Liang, Elizabeth R. Jacobs, Meetha Medhora

**Affiliations:** 1 Department of Radiation Oncology, Medical College of Wisconsin, Milwaukee, WI, USA.; 2 Department of Physiology, Medical College of Wisconsin,Milwaukee, WI, USA; 3 Department of Anesthesiology, Medical College of Wisconsin,Milwaukee, WI, USA.; 4 Cardiovascular Center, Medical College of Wisconsin, Milwaukee, WI, USA; 5 Research Service, Department of Veterans Affairs, Zablocki Veterans Affairs Medical Center, Milwaukee, WI, USA

## Abstract

We used a rat model of whole thorax x-ray irradiation to profile the microRNA (miRNA) in lung and blood up to 4 weeks after radiation. MiRNA from normal and irradiated Wistar rat lungs and whole blood were analyzed by next-generation sequencing and the changes by radiation were identified by differential deRNA-seq 1, 2, 3 and 4 weeks after irradiation. The average total reads/library was 2,703,137 with a mean of 88% mapping to the rat genome. Detailed profiles of 100 of the most abundant miRNA in rat blood and lung are described. We identified upregulation of 4 miRNA, miR-144-5p, miR-144-3p, miR-142-5p and miR-19a-3p in rat blood 2 weeks after radiation that have not previously been shown to be altered after radiation to the lung. Ingenuity Pathway Analysis identified signaling of inflammatory response pathways. These findings will support development of early detection methods, as well as mechanism(s) of injury and mitigation in patients after radiotherapy or radiological accidents.

## Radiation lung injuries

Radiation-induced lung injury is characterized by acute pneumonitis and chronic fibrosis^1^,^2^,^3^ both of which can be lethal. Acute pneumonitis in humans develops within the first 2 to 3 months after irradiation, while chronic pulmonary fibrosis manifests months or even years later^3^. We have developed a rat model of whole thoracic radiation by X-rays to induce pneumonitis from 6–12 weeks post exposure and pulmonary fibrosis after 30 weeks^4^,^5^. We and others showed early treatment with mitigators like angiotensin converting enzyme (ACE) inhibitors, enhances survival and improves lung function after radiation^6^,^7^,^8^,^9^,^10^. In fact the ACE inhibitor enalapril can be started 5 weeks after radiation to mitigate pneumonitis and fibrosis^9^. However, in the event of a radiological accident or attack, it will remain challenging to determine who to treat since accurate dosimetry may not be possible. In addition, sensitivity to radiation may vary between people. Therefore development of biomarkers to predict injuries after radiation but before symptoms develop has become an important area of research.

## Changes in miRNA associated with irradiated lungs

Circulating miRNA biomarkers have been reported in many diseases including those involving the lungs^11^. Since miRNA in circulating blood is considered to be a non-invasive measurement and can be an indication of specific disease conditions, circulating miRNA may be considered for development of biomarkers. Changes in miRNA after radiation have been reported in lung cancer patients undergoing radiotherapy in the clinic^12^. But, analysis of miRNA changes that may occur after a radiological accident or terrorism attack is not feasible in humans. Information about miRNA changes in the lung after radiation will facilitate a better understanding of the mechanism(s) of injury as well as identify molecular targets for therapy. Animal models have been used for such studies^13^,^14^. In a mouse model, Jacob *et al*. identified differentially expressed serum miRNAs 24–72 hours after total body irradiation^13^. MiRNA expression was studied using the nanostring nCounter multiplex platform, which is capable of detecting approximately 600 mouse specific miRNAs. Recently Xie *et al*. reported lung miRNA expression in response to radiation-induced lung injury in rats. Lung miRNA was evaluated with microarrays (387 miRNAs) at 3, 12 and 26 weeks. However, miRNA profiles from blood or tissues that can be obtained by minimally invasive methods (such as body fluids) were not investigated in this study^14^.

Therefore the aims of the current project were: (1) to provide comprehensive profiles of the lung and blood miRNA in rats by next-generation sequencing to detect molecular changes by radiation; (2) to look for circulating miRNAs in whole blood to develop candidate biomarkers for radiation injuries. MiRNA changes were analyzed weekly up to 4 weeks after radiation, which is one week before we can intervene to mitigate pneumonitis with an ACE inhibitor^9^, and months before pulmonary fibrosis develops.

## Results

### Total profile of miRNA from lung and blood

Total RNA was used to generate small RNA libraries (n = 36) by size selecting for miRNA (Table 1 and Methods). Each library contained miRNA pooled from 3 rats and a total of 3 libraries were analyzed for each time point 1, 2, 3 and 4 weeks after irradiation. Separate libraries were prepared from blood and lung, each represented by 9 rats/time point. The average total reads/library was 2,703,137. The average total unique reads/library was 54,268. The percent reads that mapped to the rat genome^15^ ranged from 64.7 to 98.9%, with a mean of 88%. Detailed data are shown in Table 1. A total of 947 miRNAs were identified by the next generation sequencing and 458 of them were mature unique miRNAs that have been identified in rats while 116 were homologous unique miRNAs that have been identified in other species. Another 373 miRNA were considered as new miRNA (Supplemental Table 1) that have not been previously described.

**Figure 1:**
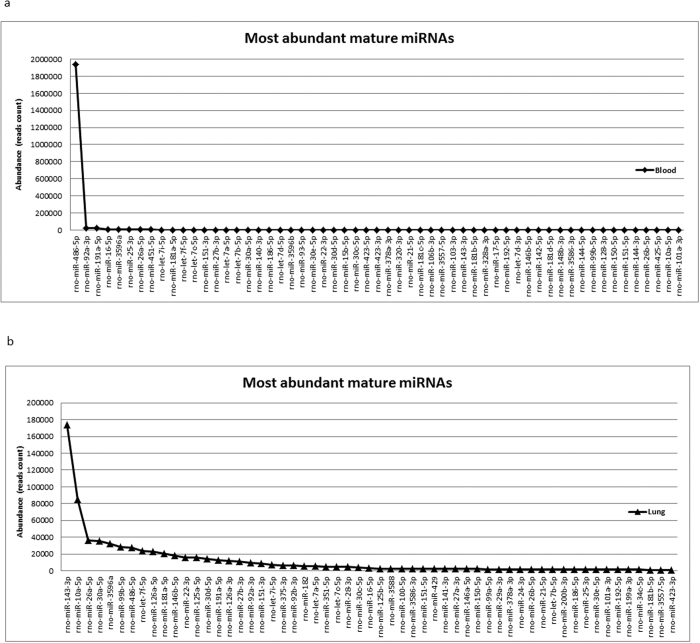
The top 57 most abundant mature miRNAs in the blood (**a**) and lung (**b**) of normal rats.

We chose to follow abundantly expressed miRNAs that had more than ~100 reads in blood and ~1000 reads in lung of the control rats (Table 2). With these thresholds, we identified 113 most expressed miRNAs in blood and lung of control rats (0 Gy) (Table 2). Among these miRNA, 43 of them from blood and 47 of them from lung were identified as new miRNAs (Table 2). In both lung and blood, 57 of the top 113 detected miRNAs were mature unique miRNAs (Fig. 1). Top 20 miRNA from lung and blood are shown in Table 3 along with results from other studies. The results from bovine blood^16^ were derived by a similar protocol as the rat blood, miRNA-seq. The rat lung miRNA in the previous study^17^ was obtained from a microarray and not by sequencing and so could be expected to show differing results. Since there was a large fold difference in expression of rno-miR-486-5p and 451-5p in rat blood but not bovine blood, we carried out RT-qPCR for a sub-set of the top 10 miRNA in whole rat blood without amplification or any additional experimental intervention. Amplification has the potential to alter ratios of expressed miRNA during library preparation. Expression of miR-486-5p by RT-qPCR was in the same range as miR-451-5p suggesting reads for miR-486-5p were detected at a much higher ratio by miRNA-seq in rat blood. The expression of miR-486-5p by RT-qPCR was found to be 9.5 fold higher than that of miR-191-5p in rats. Though miR-16-5p was not among the top 10 miRNA in bovine blood^16^ but is abundant in human erythrocytes^17^, we checked expression by RT-qPCR and found it was also in the same range as miR-451-5p and 486-5p.

### Changes in miRNA profile by radiation

The numbers of the miRNA changed after radiation in blood and lung at different time points from 1 week to 4 weeks are shown in Table 4. The total reads we identified in the lung were more than those in the blood. At 1, 2 and 3 weeks the lung was found to have more miRNAs that were changed by radiation than blood (62 vs. 2; 24 vs. 13; 22 vs. 5 increase at 1, 2 and 3 weeks respectively; 40 vs. 3; 16 vs. 3; 64 vs. 15 decrease at 1, 2 and 3 weeks respectively). However, numbers of miRNA changed at 4 weeks after radiation were similar in lung and blood (4 vs. 5 increase and 8 vs. 10 decrease). Some miRNA remained changed at more than one time-point after radiation. The numbers of the changes with overlaps at different time points of the same miRNA are also listed in the Table 4. The most overlap of the upregulated miRNA in lung was at 1 and 2 weeks, with an overlap of 11 miRNAs, while 10 miRNAs at 2 weeks and 11 at 3 weeks were also decreased at 1 week.

### Identification of circulating miRNA altered by radiation

**Figure 2:**
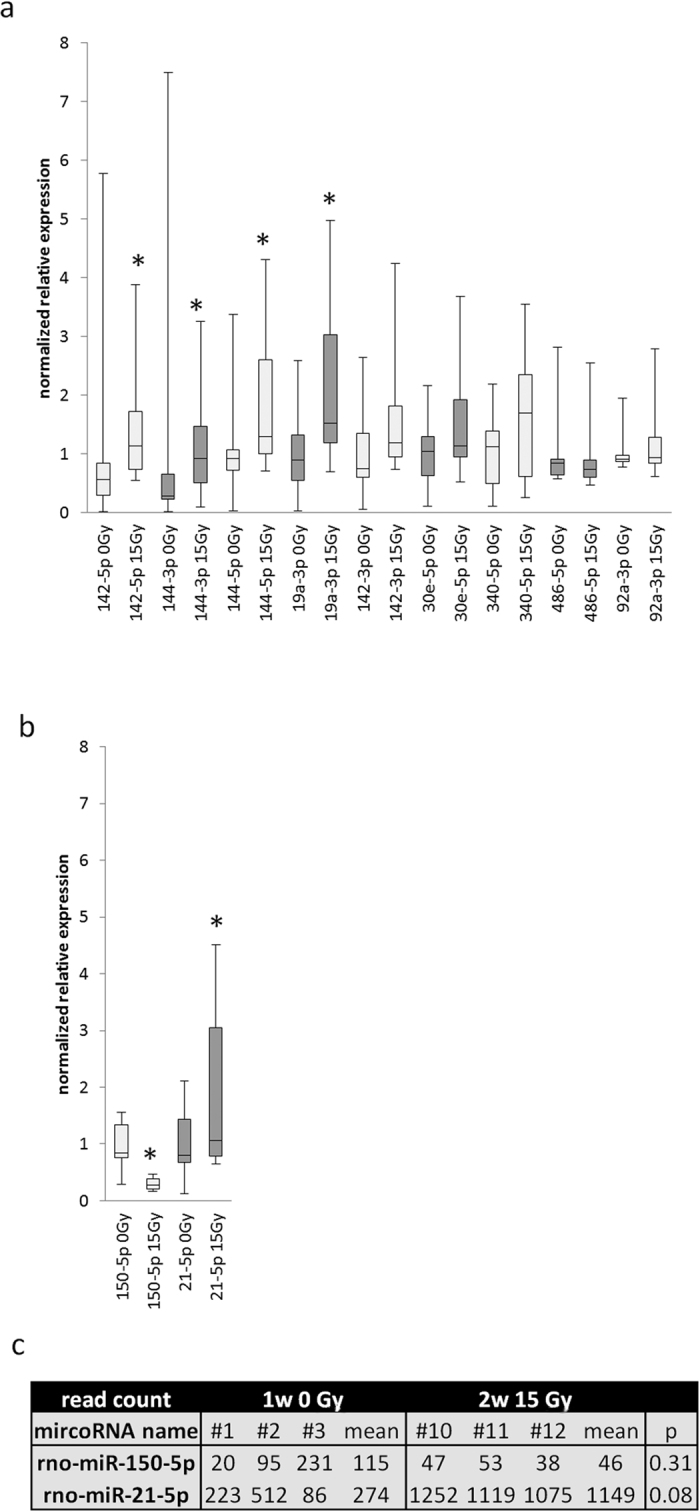
Relative miRNA expression after 2 weeks post-radiation in blood, as determined by RT-qPCR. The line in each box represents the median value. The box spans the 25th and 75th percentiles. The upper and lower bars show the maximum and minimum values. N = 11 rats/box. *P < 0.05 radiated vs. control (0 Gy). MiR-191-5p was used as reference because it shows consistent read counts in all sequenced libraries. miRNA in (**a**) were selected by results of miRNA-seq for candidates with p < 0.05 radiated vs. control by t test. miRNA in (**b**) were selected because other investigators previously identified these to be altered after radiation or lung injury. Their reads count with p values from the sequence results of this study are shown in (**c**). # refers to the library number # in Table 1.

Since the most miRNA changes after radiation in whole blood were shown to be at 2 weeks (13 increased) and 3 weeks (15 decreased) (Table 4), we focused on the 2-week time point to develop circulating miRNA markers. MiRNAs with statistical significance based on read counts from the blood libraries (p < 0.05 between 15 Gy at 2 weeks and 0 Gy at 1 week on the reads) were further tested by RT-qPCR (Fig. 2a). We focused on miRNA that increased after radiation since a decrease in miRNA may reflect the fall in circulating blood cells at 2 weeks. We verified the up regulation of these 4 miRNAs, which are miR-142-5p, 144-3p, 144-5p and 19a-3p by radiation (Fig. 2a). We also tested miRNAs by RT-qPCR that were reported by others to change after radiation^13^ and confirmed miR-150-5p was down regulated and miR-21-5p^18^ was up regulated by radiation (Fig. 2b). Sequencing results of these two miRNAs in two week blood samples followed the same trend as the RT-qPCR, though the results did not reach statistical significance between the read counts from non-irradiated controls (0 Gy) versus 15 Gy. The read counts are given in Fig. 2c. All the miRNA that we verified to be changed by RT-qPCR at 2 weeks after radiation in blood were further tested in the lung tissue at 2 weeks. Except 150-5p which showed the same change (decreased after radiation), none of them were changed in lung (data not shown). Other miRNAs described in the literature, but which did not show changes in our rat model in blood between controls and radiated rats at 2 weeks were 99b-5p, 30a-5p, let7-5p 9a-5p and 92b-3p (data not shown). The results of the RT-qPCR on these miRNAs matched our findings from sequencing.

### Changes in circulating blood cells after whole thorax irradiation

**Figure 3:**
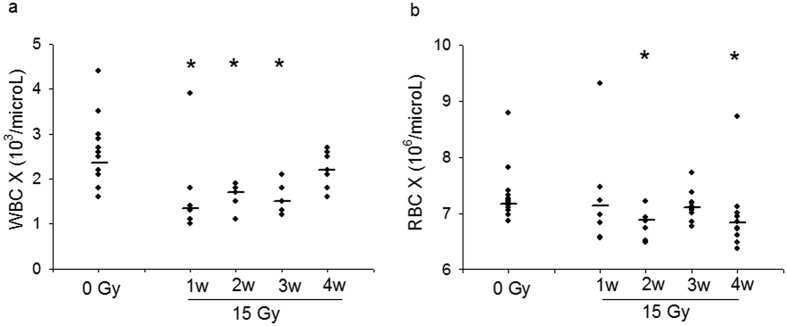
Blood cell counts versus time are shown (**a**) White blood cell count (WBC) and (**b**) Red blood cell count (RBC). *p < 0.05 vs. control (0 Gy). N = 14 (controls), 6 (irradiated at 1 week), 7 (irradiated at 2 weeks), 9 (irradiated at 3 weeks), 9 (irradiated at 4 weeks). The line represents the median.

The WBC are lower after one week while RBC fall by 2 weeks after radiation (Fig. 3). The WBC recover by 4 weeks.

### Pathway analysis of miRNA changes in blood

**Figure 4:**
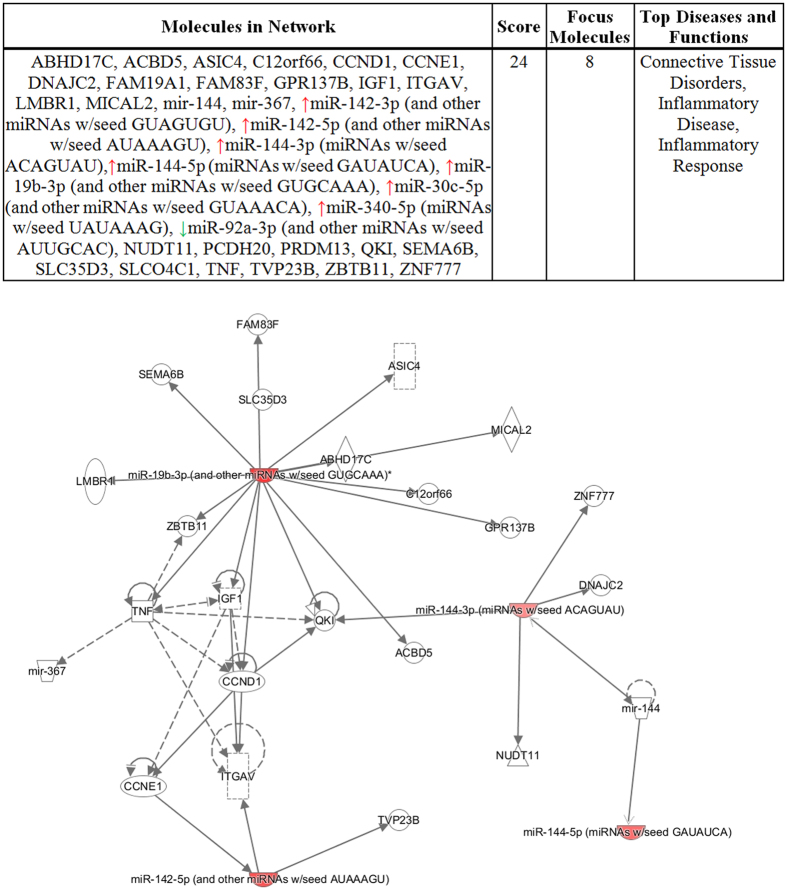
Ingenuity pathway analysis of the sequence data showed the top scoring diseases and function that are associated with our model (upper panel). The pathway map is presented below and miRNA that were significantly changed in the miRNA-seq study and also validated by qPCR are shown as highlighted in red.

The changes observed in the 2 week sequencing data from blood were also used to explore signaling pathways that are altered by radiation using the Ingenuity Pathway Analysis platform. The results showed that the top diseases and functional pathways that can be associated with the blood at 2 weeks after radiation were inflammatory disease, inflammatory response and connective tissue disorders (Fig. 4).

## Discussion

In this study, we report the miRNA profile obtained by RNA-seq from rat lung and blood at baseline and 1, 2, 3 and 4 weeks after 15 Gy whole thorax irradiation. This dose induces lethal pneumonitis in rats after 42 days, though all rats survive 12 Gy whole thorax irradiation^19^. Doses of 13 or 14 Gy induce intermediate levels of lethality^19^. We used RT-qPCR to verify changes in levels of at least 6 circulating miRNA that were identified by RNA-seq at 2 weeks after 15 Gy. At this time point all rats are healthy with normal breathing rates and lung histology though we have measured an increase in vascular permeability and apoptosis in the radiated lungs^20^. Some of the miRNA that were altered in the circulation e.g. miRs 142-5p, 150-5p and 21-5p have been described in anti-apoptotic responses and may signify cellular compensation for increased hematopoietic death induced by radiation^21^,^22^,^23^,^24^.

The miRNA molecules we sequenced were sized by gel purification to exclude RNA other than miRNA. Our results show miRNA-seq to be a sensitive technique, yielding a dynamic range of read counts from 1 − 1.9 × 10^6^ (Supplemental Table 1). Additionally 88 and 98% mean reads mapped to the rat genome in the lung and blood samples respectively (Table 1). Unmapped reads could occur by sequencing errors, artifacts during PCR amplification, short reads, trimming or repetitive DNA sequences etc. Most changes in miRNA were observed at 1 week after radiation in lung tissue. MiRNA changes at earlier time points have been evaluated in other models of radiation but not with exhaustive techniques such as miRNA-seq.

High-throughput technology such as miRNA-seq has many advantages including generation of large data sets which reflect the biology of the system. However, our study, in which expression of miR-486-5p may be overrepresented, also demonstrates limitations. Though most of the top 10 miRNA species which we identified as expressed in whole blood corresponds with another report^16^, expression of miRNA of interest should be confirmed by independent techniques/platforms regardless of specific technology or methods. We validated candidate circulating miRNAs from the high throughput RNA-seq individually by RT-qPCR. Nine miRNA showing increase from control after radiation were selected for verification by RT-qPCR. Though most miRNA trended in the same direction in both assays only 4 miRNA were statistically increased after RT-qPCR.

The profile of miRNA in normal rat blood and lung (0 Gy, without radiation) that we report in this paper (Tables 2and 3, Fig. 1) and elaborated in the supplement, shows new species that fit the definition of miRNA but remain to be characterized. More than 100 miRNA from blood and 1000 from lungs of non-irradiated rats yielded read counts greater than 100 (a value that can be consistently and easily verified by RT-qPCR). Both blood and lung tissue had 57 mature miRNAs that have been previously identified in the top 113 detected (Fig. 1). Consistent with the previous findings^25^, high expression of miR-486-5p in red blood cells was also detected in whole rat blood. This miRNA was described to regulate normal erythropoiesis^26^ and was also suggested to be an important oncogene in several hematopoietic myeloid malignancies^26^. MiR-486-5p was reported to be a potential plasma-based biomarker for lung cancer^27^. Leuenberge *et al*. showed that its abundance remained unchanged after blood transfusion in healthy males^28^. Therefore, it was used as an endogenous control for data normalization in blood miRNA studies^28^,^29^. In the current study, we observed decrease in miR-486-5p read-counts in rat blood at 2 weeks after radiation. However these changes could not be confirmed by RT-qPCR (Fig. 2). This, along with verification of only 4/9 candidate miRNA by RT-qPCR reiterates the limitation of miRNA-seq.

Using next generation sequencing, we report changing miRNA patterns after radiation in rat lung and blood (Table 4). All RNA used for these comparisons were prepared at the same time with identical reagents and equipment. Such data can provide useful algorithms to develop early biomarkers for treatment plans in radiotherapy. As an adjunct diagnostic method, next generation sequencing provides abundant information. We verified an increase of miR-142-5p, 144-3p, 144-5p and 19-3p in blood after 15 Gy in our rat model. Another two interesting miRNA (miR-150-5p and 21-5p) were selected for testing by RT-qPCR based on changes after radiation as reported by others. MiR-150-5p was shown to be decreased after radiation in a mouse model^13^. It is also expressed in blood and hematopoietic cells, which, as mentioned, are sensitive to radiation, explaining the fall in circulating levels of this miRNA after 15 Gy whole thorax irradiation. MiR-21-5p is a well-known lung injury marker^30^,^31^,^32^. But studies with changes in miR-21-5p after radiation have been limited to lung cancer cells^23^,^33^ and not in irradiated lung tissue. However some non-radiation studies have associated changes in this microRNA. Circulating miR-21 was suggested by Loboda *et al*. to be a potential early biomarker of renal fibrosis, because it was shown that upregulation of miR-21 alters metabolic pathways and leads to renal fibrosis^34^. MiR-21 was also suggested to plays a dynamic role in inflammatory responses^34^. Since inflammation and fibrosis are considered as major radiation responses in many organs including lungs, it is possible that miR-21 also plays some roles in radiation response in normal tissues. Beside circulation miR-21, exosomal miR-21 was also suggested to have a strong potential to be used as a universal biomarker to identify cancers^35^. In fact, based on one report, exosomal miR-21 seemed to be better than circulating miR-21 to serve as such a biomarker^35^. We confirmed a decrease in 150-5p and increase in 21-5p after radiation by RT-qPCR in whole blood from our rat model of thorax radiation. Though these miRNA trended in the same direction with RNA-seq data, there was considerable variation between samples in the libraries, so that the results were not statistically significant. As already mentioned, since the libraries were amplified and pooled these variations could be artifacts. We focused on the changes of miRNA at 2 weeks, since not only as mentioned, that was the time when the most increases in miRNA were detected, but also when most of the mature miRNA were found to be changed after radiation. In addition, we have also established a SPECT/CT biomarker to predict radiation lung injuries^36^. By this method, the most significant lung injuries, namely the pulmonary cell death measured by 99mTc-labeled Duramycin and pulmonary vascular resistance and vascular permeability measured in isolated perfused lungs were found to be at 2 weeks after 15 Gy in the same rat model.

MiR-144 has been reported to affect sensitivity in radiotherapy by promoting proliferation, migration and invasion of breast cancer cells^37^. Dysregulation of miR-144 has been described in studies of multiple cancers^38^,^39^,^40^,^41^. It was suggested to be a tumor suppressor by Matsushita *et al*.^38^ and inhibited proliferation but promoted apoptosis and autophagy through targeting the p53-induced glycolysis and apoptosis regulator TIGAR^40^. Besides the current study, elevation of miR-144 was also reported in many non-cancer studies^42^,^43^. MiR-144 is overexpressed in peripheral blood mononuclear cells from patients with pulmonary tuberculosis (TB) and regulated anti-TB immune response in T cells^42^. Hassan *et al*. found increase in miR-144 in human bronchial epithelial (HBE) cells exposed to cigarette smoke extract and cadmium. Su *et al*. showed that miR-144 regulates hematopoiesis and vascular development by repressing expression of meis1 in zebrafish^44^. In summary miR-144 plays a role in inflammation, immune response and suppression of cell growth, processes that are activated by radiation. It is surprising that we observed increase in this miRNA in whole blood, since circulating cells are decreased by radiation and this miRNA was not increased in irradiated lungs in our model.

MiR-19a-3p has been reported to inhibit breast cancer progression and metastasis by inducing macrophage polarization^45^. It was also suggested to be involved in inflammatory processes by directly regulating 5-LO (5-lipoxygenase) expression in T lymphocytes^46^. Busch *et al*. found that the inhibition of miR-19a-3p with an antagomir led to an increase in 5-LO mRNA expression in T lymphocytes^46^. We speculate that this miRNA may be induced in response to inflammatory signaling after radiation to down-regulate lipoxygenase metabolites in blood cells such as macrophages and T-lymphocytes.

MiR-142-5p has also been demonstrated to be involved in inflammation^21^,^22^,^47^. Su *et al*. showed miR-142-5p regulates human and mouse macrophage profibrogenic gene expression in chronic inflammation and models of liver and lung fibrosis^47^. Increases of miR-142-5p were found in lungs of patients with idiopathic pulmonary fibrosis^47^. MiR-142-5p was reported to control T-cell responses *in vitro* and in murine models of graft versus host disease^22^. Finally miR-142-5p had tumor-suppressive effect in lung cancer cells^48^,^49^. In summary, similar to miR-144, miR-142-5p regulates cell growth and inflammation. Interestingly miR-142-5p expression in macrophages also influences fibrosis in the lung^47^, a phenotype that is induced by radiation. It is possible that detection of increase in this miRNA in spite of a decrease in circulating cells in the blood at 2 weeks after radiation suggests regulation of genes that could lead to the later effects of radiation. Perhaps these immune cells may be responsible for radiation pulmonary injury after infiltration into the lungs.

We also conducted pathway analysis to determine signaling changes in the lung after radiation, based on the changes in miRNA as determined by miRNA-seq. The pathways with the highest scores by Ingenuity Pathway Analysis were “cancer, organismal injury” and “abnormalities and reproductive system disease”, while inflammatory responses received a lower score (results not shown). Pathways derived from changes that we confirmed in the blood of irradiated rats are shown in Fig. 4. To our knowledge this is the first time that miR-144-3p, 144 -5p, and 19a-3p are upregulated in rat blood 2 week after irradiation of the thorax. Their function in inflammatory responses is suggested by the Ingenuity Pathway Analysis (Fig. 4). These miRNA were not changed in the irradiated lung, strongly suggesting they may be upregulated in other cells or tissues. We cannot rule out their presence in exosomes from endothelial cells within the lung. They could also be present in exosomes from other irradiated organs, e.g. the heart, partial bone marrow and blood within the thorax, that were in the field of radiation. We know immune cells are involved in radiation-pneumonitis with inflammatory infiltrates detected in the lung by histology and other diagnostic methods^50^,^51^. Vascular damage and remodeling are also found during radiation pneumonitis in human and animal lungs^51^,^52^.

Decreases in numbers of circulating blood cells are anticipated and were measured after whole thorax irradiation due to the volume of bone marrow in the field of exposure. WBC have shorter half-lives than RBC so their numbers fall before the RBC. The numbers of WBC recover by 4 weeks. We have observed increases in circulating miRNA we report after radiation, which could also be due to increase in specific transcription induced by radiation in blood cells, but not from changes in numbers of circulating cells, since these numbers were lower at the time points we examined. Future investigation with models involving radiation to the lower hemi-body or heart alone will help to highlight the effect of radiation to the lung on these miRNA changes.

## Methods

### Animal care and irradiation

All animal protocols and euthanasia criteria were approved by the Institutional Animal Care and Use Committee (IACUC). All methods were performed in accordance to the IACUC guidelines and regulations. Radiation was performed as described previously^53^. In brief, un-anesthetized 9- to 10-week-old female WAG/RijCmcr rats weighing approximately 140 g were immobilized in a plastic jig and irradiated with 320-kVp orthovoltage system X-rays, with a half-value layer (HVL) of 1.4 mm Cu. Rats were treated with a single dose of 15 Gy to the whole thorax at dose rate of 1.43 Gy/min. The radiation dose was delivered by two equally-weighted lateral beams to improve uniformity. The whole lung, heart and a small amount of liver were in the field. One group of age-matched rats was not irradiated (non-irradiated controls or 0 Gy group) but maintained under identical conditions. Experiments were terminated at 1, 2, 3 or 4 weeks. Age-matched controls were included at the 1 and 4 week time-points.

### RNA isolation

Lung tissue from the right superior lobe was cut and ~100 mg was weighed and immersed in TRIzol Reagent (Ambion/RNA by Life Technologies) immediately after sacrifice. Whole blood (0.5 ml) collected by cardiac puncture in an EDTA coated needle and syringe was directly used to extract RNA immediately without any further processing. TRIzol Reagent (Ambion/RNA (Life Technologies)) was used. Total RNA from lung and whole blood were isolated following the protocol provided by the manufacturer. RNA was stored at −80°C for future use.

### Small RNA library preparation and sequencing

Total extracted RNA was quantified using Nanodrop 2000 (Thermo Scientific). A_260/280_ was used to ensure the RNA quality. Equal amounts of RNA from three rats were pooled. The range of A_260/280_ was maintained between 1.6 and 2.1. Samples with ratios below 1.6 were discarded. Small RNA libraries (36 totals) were generated with the TruSeq Small RNA Library Prep kit (Illumina) following the manufacture’s instruction with minor changes (Table 1). In brief, 1 μg of the pooled total RNA was ligated with 3′ and 5′ adapters. Reverse transcription followed by PCR (based on the 3′ and 5′ adapter sequences for 16 cycles) was used to create cDNA. The amplified cDNA was purified using 6% PAGE Gel and bands between 147nt and 157nt which contains RNA fragment of 22nt and 30nt corresponding to miRNA were cut out using size markers and concentrated by ethanol precipitation. Libraries were visualized and quantitated with Agilent Technologies 2100 Bioanalyzer using DNA-1000 Chip. Next generation sequencing was performed at the Human and Molecular Genetics Center Sequencing Core at MCW^15^. In brief, prepared libraries were loaded onto the flow cell using a cBot instrument (Illumina) and clusters were generated in the flow cell after a 4-hour-PCR amplification. The flow cell was loaded onto the HiSeq 2000 sequencer. The obtained reads were fed into the in-house CASAVA informatics pipeline, where they were de-multiplexed and aligned to a reference genome miRbase v19.

### Analysis of small RNA deep sequencing data

Before the analysis, the adapter sequences were first removed from the output sequence reads by the tool “trim galore”^54^. Sequences with low quality (base quality < 13) at both ends of reads were further trimmed by the FastQC^55^ and mapped against miRBase v19 to identify known miRNAs using Bowtie^56^, including rat miRNAs and homologs of miRNAs known in species other than the rat. Sequence reads that did not map to miRBase were then mapped against mRNA database, Rfam (for other noncoding RNA), and RepBase (for repetitive elements) to remove reads corresponding to transcribed sequences that were not miRNAs. The remaining reads were used to predict new miRNAs with miRanalyzer^57^. miRanalyzer employs a machine learning approach based on the random forest method. With the default parameter setting, miRanalyzer can obtain the area under the curve value of 97.9% with a true positive rate of 0.79 and a false positive rate of 0.007 for predicting new mammalian miRNAs. To normalize and test differential expression, we used number of reads of known and newly identified miRNAs as input for the Bioconductor DESeq package^58^. DESeq uses a negative binomial distribution to model reads of miRNAs and to test for differential expression in deep sequencing datasets. The Benjamini-Hochberg method was used to control false discovery rate (FDR) in all statistical tests^59^.

### Real-time quantitative RT-qPCR

Validation of changes in miRNA expression of selected markers was performed by RT-qPCR from the blood of rats used for miRNA-seq as well as from independently irradiated and control rats. Total RNA from each rat were required to meet the following quality criteria: 1) A_260/280_ > 1.6, 2) yield of RNA was enough to carry out all the RT-qPCR reactions needed for all the primers tested. There was no pre-amplification step prior to PCR. LNA-primers were obtained from Exiqon and reactions were carried out after reverse transcription using miRCURY LNA^TM^ Universal RT microRNA PCR kit from Exiqon with sybr green mastermix from Biotool. The relative expression of each miRNA to a reference miR-191-5p was calculated by the formula: 2^−∆CT^ (∆C_T=_C_T_ (target-reference). Data was then normalized to the mean relative expression of controls, to determine the fold change after radiation. MiR-191-5p was used as reference for normalization because it shows consistent read counts in the libraries by sequencing.

### Blood counts

Blood was collected by cardiac puncture into EDTA tubes. White blood cell and red blood cell counts were performed by the Marshfield Laboratories the same day after checking for adequate volume and absence of clots (Marshfield, WI).

### Statistical analysis

Sigmaplot software was used to perform statistical analysis on the RT-qPCR results and the blood cell count results. For RT-qPCR, t test was used on 19a-3p and 21-5p; Mann-Whitney U test was used on 142-5p, 144-3p, 144-5p and 150-5p, when the data failed either normality test or equal variance test. For blood cell count, Kruskal-Wallis One Way Analysis of Variance on Ranks Multiple Comparisons versus Control Group (0 Gy) was conducted. Dunn’s method was used as post hoc test.

## Additional Information

**How to cite this article:** Gao, F. *et al*. Changes in miRNA in the lung and whole blood after whole thorax irradiation in rats. *Sci. Rep.* **7**, 44132; doi: 10.1038/srep44132 (2017).

**Publisher's note:** Springer Nature remains neutral with regard to jurisdictional claims in published maps and institutional affiliations.

## Supplementary Material

Supplementary Table 1

## Figures and Tables

**Table 1:** 36 libraries (18 from lung and 18 from blood) were generated.

Library #	Tissue	Radiation	Timepoint	total unique reads	total reads	unmapped unique reads	unmapped reads	%mapped reads
1	Blood	0 Gy	1 week	27983	4263244	7279	47509	98.9
2	Blood	0 Gy	1 week	38512	3443368	10356	52807	98.5
3	Blood	0 Gy	1 week	30772	4415596	8186	59870	98.6
4	Blood	0 Gy	4 weeks	32410	4019965	8521	54521	98.6
5	Blood	0 Gy	4 weeks	34329	4607361	9001	64078	98.6
6	Blood	0 Gy	4 weeks	50163	4228658	12766	77878	98.2
7	Blood	15 Gy	1 week	33005	4188089	8374	50762	98.8
8	Blood	15 Gy	1 week	34273	3477659	8872	52756	98.5
9	Blood	15 Gy	1 week	38374	3784442	9743	51171	98.6
10	Blood	15 Gy	2 weeks	32833	2257353	7819	38353	98.3
11	Blood	15 Gy	2 weeks	39243	3458791	9858	71994	97.9
12	Blood	15 Gy	2 weeks	40031	3490172	10290	74264	97.9
13	Blood	15 Gy	3 weeks	24835	3917978	6714	43091	98.9
14	Blood	15 Gy	3 weeks	37330	4006866	9804	70381	98.2
15	Blood	15 Gy	3 weeks	32440	3533270	8361	45112	98.7
16	Blood	15 Gy	4 weeks	34195	3265611	9001	65210	98.0
17	Blood	15 Gy	4 weeks	42711	3665273	11068	62775	98.3
18	Blood	15 Gy	4 weeks	25427	3361941	6713	35690	98.9
19	Lung	0 Gy	1 week	87819	2066852	20810	470429	77.2
20	Lung	0 Gy	1 week	68449	1574877	16564	501396	68.2
21	Lung	0 Gy	1 week	4796	25166	1102	4535	82.0
22	Lung	0 Gy	4 weeks	147735	4586020	36274	803019	82.5
23	Lung	0 Gy	4 weeks	138509	3087369	34716	803220	74.0
24	Lung	0 Gy	4 weeks	71645	1882795	17737	219969	88.3
25	Lung	15 Gy	1 week	99936	2051176	24196	356010	82.6
26	Lung	15 Gy	1 week	40336	653589	11873	223218	65.8
27	Lung	15 Gy	1 week	96498	2195629	22424	290730	86.8
28	Lung	15 Gy	2 weeks	95576	1739850	26142	153659	91.2
29	Lung	15 Gy	2 weeks	61195	1175767	16564	300830	74.4
30	Lung	15 Gy	2 weeks	33465	496463	9820	175151	64.7
31	Lung	15 Gy	3 weeks	93148	1536333	25929	413899	73.1
32	Lung	15 Gy	3 weeks	82228	1883847	19744	295489	84.3
33	Lung	15 Gy	3 weeks	4076	23541	1536	7505	68.1
34	Lung	15 Gy	4 weeks	106685	3150602	26561	804574	74.5
35	Lung	15 Gy	4 weeks	62832	1379394	17365	406278	70.5
36	Lung	15 Gy	4 weeks	29857	418039	9462	123817	70.4
**Mean**				54268	2703137	13932	204776	88

Each library contained microRNA from 3 rats and 3 libraries were analyzed at each time point, representing 9 rats/time point. Read count and mapping rate to the rat genome of each library are shown.

**Table 2:** The 113 most highly expressed microRNAs in blood and lung of control rats (0 Gy) were listed.

Most highly expressed (top 113) miRNA							
rat blood	category	read counts (n = 6)	% top 113	rat lung	category	read counts (n = 6)	% top 113
rno-miR-486-5p	mature_unique	1940840	92.566	chr18_57625094_57625241	newMicroRNA_genome	207773	12.896
rno-miR-92a-3p	mature_unique	23299	1.111	chr18_57625124_57625204	newMicroRNA_fastgenome	207425	12.875
rno-miR-191a-5p	mature_unique	21565	1.029	rno-miR-143-3p	mature_unique	174207	10.813
rno-miR-16-5p	mature_unique	9957	0.475	rno-miR-10a-5p	mature_unique	84796	5.263
chr8_123981613_123981719	newMicroRNA_homolog	7772	0.371	chr8_123981613_123981719	newMicroRNA_homolog	42502	2.638
bta-miR-26c	maturehomolog_unique	7370	0.351	chr8_123981623_123981705	newMicroRNA_fasthomolog	42468	2.636
rno-miR-3596a	mature_unique	7150	0.341	bta-miR-26c	maturehomolog_unique	41087	2.550
rno-miR-25-3p	mature_unique	6881	0.328	rno-miR-26a-5p	mature_unique	36273	2.251
rno-miR-26a-5p	mature_unique	6610	0.315	rno-miR-30a-5p	mature_unique	35274	2.189
rno-miR-451-5p	mature_unique	4558	0.217	rno-miR-3596a	mature_unique	32088	1.992
rno-let-7i-5p	mature_unique	3874	0.185	chr1_56486962_56487048	newMicroRNA_genome	31215	1.937
rno-miR-181a-5p	mature_unique	3794	0.181	chr9_22142880_22142968	newMicroRNA_fasthomolog	31181	1.935
rno-let-7f-5p	mature_unique	3021	0.144	chr9_22142869_22142978	newMicroRNA_homolog	31181	1.935
chr18_8923954_8924050	newMicroRNA_genome	2936	0.140	chr1_56486961_56487047	newMicroRNA_fastgenome	29074	1.805
rno-let-7c-5p	mature_unique	2780	0.133	rno-miR-99b-5p	mature_unique	28132	1.746
chr17_7351428_7351565	newMicroRNA_genome	2508	0.120	rno-miR-486-5p	mature_unique	27940	1.734
chrX_139740786_139740910	newMicroRNA_genome	2394	0.114	rno-let-7f-5p	mature_unique	24028	1.491
rno-miR-151-3p	mature_unique	2393	0.114	rno-miR-126a-5p	mature_unique	22723	1.410
rno-miR-27b-3p	mature_unique	2224	0.106	rno-miR-181a-5p	mature_unique	20558	1.276
rno-let-7a-5p	mature_unique	1291	0.062	chr1_251558040_251558146	newMicroRNA_genome	19452	1.207
chr9_22142880_22142968	newMicroRNA_fasthomolog	1123	0.054	chr1_251558049_251558132	newMicroRNA_fastgenome	19452	1.207
chr9_22142869_22142978	newMicroRNA_homolog	1123	0.054	bta-miR-3600	maturehomolog_unique	18827	1.169
rno-let-7b-5p	mature_unique	1112	0.053	chr10_62782697_62782840	newMicroRNA_homolog	18640	1.157
bta-miR-3596	maturehomolog_unique	1071	0.051	chr10_62782728_62782812	newMicroRNA_fasthomolog	18623	1.156
rno-miR-30a-5p	mature_unique	1041	0.050	rno-miR-146b-5p	mature_unique	17794	1.104
chr19_37422692_37422817	newMicroRNA_genome	979	0.047	chr1_56487554_56487694	newMicroRNA_genome	17044	1.058
chr19_37422714_37422799	newMicroRNA_fastgenome	979	0.047	rno-miR-22-3p	mature_unique	16162	1.003
rno-miR-140-3p	mature_unique	876	0.042	rno-miR-125a-5p	mature_unique	15567	0.966
rno-miR-186-5p	mature_unique	812	0.039	rno-miR-30d-5p	mature_unique	13955	0.866
rno-let-7d-5p	mature_unique	809	0.039	rno-miR-191a-5p	mature_unique	12568	0.780
rno-miR-3596b	mature_unique	801	0.038	chr17_7351428_7351565	newMicroRNA_genome	11758	0.730
rno-miR-93-5p	mature_unique	770	0.037	rno-miR-126a-3p	mature_unique	11732	0.728
hsa-miR-148a-3p	maturehomolog_unique	657	0.031	chr17_7351451_7351539	newMicroRNA_fastgenome	11136	0.691
rno-miR-30e-5p	mature_unique	642	0.031	chr1_56487580_56487664	newMicroRNA_fastgenome	11053	0.686
bta-miR-3600	maturehomolog_unique	621	0.030	bta-miR-2340	maturehomolog_unique	11037	0.685
chr10_62782697_62782840	newMicroRNA_homolog	613	0.029	rno-miR-27b-3p	mature_unique	10674	0.662
chr17_7351451_7351539	newMicroRNA_fastgenome	542	0.026	rno-miR-92a-3p	mature_unique	9513	0.590
rno-miR-22-3p	mature_unique	538	0.026	chr7_105819723_105819875	newMicroRNA_genome	9073	0.563
rno-miR-30d-5p	mature_unique	525	0.025	rno-miR-151-3p	mature_unique	8838	0.549
chr7_105819723_105819875	newMicroRNA_genome	497	0.024	chr9_74233487_74233589	newMicroRNA_genome	7084	0.440
rno-miR-15b-5p	mature_unique	471	0.022	rno-let-7i-5p	mature_unique	6882	0.427
chr10_67078689_67078839	newMicroRNA_homolog	453	0.022	rno-miR-375-3p	mature_unique	6440	0.400
chr10_67078715_67078809	newMicroRNA_fasthomolog	453	0.022	rno-miR-92b-3p	mature_unique	6216	0.386
hsa-miR-3184-3p	maturehomolog_unique	447	0.021	rno-miR-182	mature_unique	5300	0.329
rno-miR-30c-5p	mature_unique	438	0.021	rno-let-7a-5p	mature_unique	5173	0.321
rno-miR-423-5p	mature_unique	412	0.020	rno-miR-351-5p	mature_unique	5141	0.319
hsa-miR-103b	maturehomolog_unique	404	0.019	rno-let-7c-5p	mature_unique	5023	0.312
rno-miR-423-3p	mature_unique	398	0.019	rno-miR-28-3p	mature_unique	4798	0.298
rno-miR-378a-3p	mature_unique	391	0.019	chr2_181395394_181395545	newMicroRNA_genome	3728	0.231
chr3_118996576_118996714	newMicroRNA_homolog	389	0.019	rno-miR-30c-5p	mature_unique	3643	0.226
chr10_20694998_20695142	newMicroRNA_homolog	389	0.019	rno-miR-16-5p	mature_unique	3389	0.210
chr19_25667395_25667479	newMicroRNA_fastgenome	389	0.019	chr2_181395424_181395508	newMicroRNA_fastgenome	3346	0.208
rno-miR-320-3p	mature_unique	372	0.018	chr19_25638547_25638840	newMicroRNA_genome	3206	0.199
chr15_50842920_50843068	newMicroRNA_genome	358	0.017	chr8_44518661_44518763	newMicroRNA_homolog	2946	0.183
rno-miR-21-5p	mature_unique	341	0.016	rno-miR-125b-5p	mature_unique	2753	0.171
chr10_74864475_74864611	newMicroRNA_genome	333	0.016	rno-miR-3588	mature_unique	2703	0.168
chr10_76049262_76049384	newMicroRNA_genome	325	0.015	chr11_78139638_78139790	newMicroRNA_homolog	2644	0.164
chr10_76049282_76049366	newMicroRNA_fastgenome	320	0.015	chr11_78139668_78139752	newMicroRNA_fasthomolog	2644	0.164
chr5_137468994_137469092	newMicroRNA_genome	319	0.015	ppy-miR-151b	maturehomolog_unique	2623	0.163
rno-miR-181c-5p	mature_unique	305	0.015	rno-miR-100-5p	mature_unique	2588	0.161
bta-miR-2340	maturehomolog_unique	304	0.014	rno-miR-3586-3p	mature_unique	2572	0.160
rno-miR-106b-3p	mature_unique	300	0.014	hsa-miR-148a-3p	maturehomolog_unique	2551	0.158
rno-miR-3557-5p	mature_unique	300	0.014	chr19_25638578_25638661	newMicroRNA_fastgenome	2535	0.157
chr12_17608366_17608479	newMicroRNA_genome	291	0.014	chr5_172897165_172897291	newMicroRNA_genome	2432	0.151
chr12_17608379_17608463	newMicroRNA_fastgenome	291	0.014	rno-miR-151-5p	mature_unique	2427	0.151
rno-miR-103-3p	mature_unique	288	0.014	rno-miR-429	mature_unique	2358	0.146
chr3_18651980_18652124	newMicroRNA_genome	264	0.013	rno-miR-141-3p	mature_unique	2308	0.143
rno-miR-143-3p	mature_unique	243	0.012	chr1_95595998_95596190	newMicroRNA_genome	2214	0.137
rno-miR-181b-5p	mature_unique	243	0.012	chr1_95596079_95596160	newMicroRNA_fastgenome	2214	0.137
chr15_99853725_99853834	newMicroRNA_genome	234	0.011	rno-miR-27a-3p	mature_unique	2197	0.136
rno-miR-328a-3p	mature_unique	226	0.011	rno-miR-146a-5p	mature_unique	2142	0.133
rno-miR-17-5p	mature_unique	225	0.011	rno-miR-150-5p	mature_unique	2075	0.129
cgr-miR-1285	maturehomolog_unique	222	0.011	chr9_73734035_73734173	newMicroRNA_genome	1968	0.122
chr2_255655012_255655108	newMicroRNA_genome	221	0.011	chr11_16397521_16397651	newMicroRNA_homolog	1963	0.122
rno-miR-192-5p	mature_unique	210	0.010	chr11_78139643_78139779	newMicroRNA_genome	1940	0.120
chrX_139741405_139741553	newMicroRNA_genome	208	0.010	chr11_78139668_78139752	newMicroRNA_fastgenome	1937	0.120
hsa-miR-3184-5p	maturehomolog_unique	205	0.010	chr13_77910750_77910898	newMicroRNA_homolog	1889	0.117
chr1_209040238_209040380	newMicroRNA_genome	192	0.009	chr13_77910785_77910868	newMicroRNA_fasthomolog	1889	0.117
chr18_57625094_57625241	newMicroRNA_genome	188	0.009	rno-miR-99a-5p	mature_unique	1817	0.113
bta-miR-2487	maturehomolog_unique	187	0.009	rno-miR-29a-3p	mature_unique	1789	0.111
rno-let-7d-3p	mature_unique	182	0.009	chr9_22142881_22142971	newMicroRNA_fastgenome	1760	0.109
chr19_25667542_25667672	newMicroRNA_genome	179	0.009	rno-miR-378a-3p	mature_unique	1752	0.109
chr1_251558040_251558146	newMicroRNA_genome	177	0.008	rno-miR-24-3p	mature_unique	1750	0.109
rno-miR-146b-5p	mature_unique	175	0.008	rno-miR-26b-5p	mature_unique	1743	0.108
bta-miR-2898	maturehomolog_unique	169	0.008	rno-miR-21-5p	mature_unique	1699	0.105
chr1_95595998_95596190	newMicroRNA_genome	168	0.008	bta-miR-3604	maturehomolog_unique	1692	0.105
rno-miR-142-5p	mature_unique	168	0.008	chr10_74864475_74864611	newMicroRNA_genome	1682	0.104
rno-miR-181d-5p	mature_unique	166	0.008	rno-let-7b-5p	mature_unique	1671	0.104
chr1_95596079_95596160	newMicroRNA_fastgenome	164	0.008	chr9_22142873_22142980	newMicroRNA_genome	1663	0.103
rno-miR-148b-3p	mature_unique	164	0.008	rno-miR-200b-3p	mature_unique	1616	0.100
rno-miR-3586-3p	mature_unique	162	0.008	chr5_172898998_172899090	newMicroRNA_genome	1609	0.100
rno-miR-144-5p	mature_unique	161	0.008	rno-miR-186-5p	mature_unique	1563	0.097
rno-miR-99b-5p	mature_unique	161	0.008	rno-miR-25-3p	mature_unique	1555	0.097
rno-miR-128-3p	mature_unique	161	0.008	rno-miR-30e-5p	mature_unique	1546	0.096
rno-miR-150-5p	mature_unique	159	0.008	rno-miR-101a-3p	mature_unique	1450	0.090
rno-miR-151-5p	mature_unique	153	0.007	rno-miR-192-5p	mature_unique	1434	0.089
chr8_116727221_116727334	newMicroRNA_genome	150	0.007	chr3_57340837_57340983	newMicroRNA_genome	1427	0.089
rno-miR-144-3p	mature_unique	149	0.007	chr3_57340843_57340973	newMicroRNA_homolog	1403	0.087
rno-miR-26b-5p	mature_unique	147	0.007	rno-miR-199a-3p	mature_unique	1381	0.086
rno-miR-425-5p	mature_unique	142	0.007	rno-miR-34c-5p	mature_unique	1319	0.082
chr19_35122527_35122673	newMicroRNA_genome	134	0.006	chr8_54422016_54422156	newMicroRNA_genome	1279	0.079
chr1_56486961_56487047	newMicroRNA_fastgenome	132	0.006	chr8_54422050_54422129	newMicroRNA_fastgenome	1277	0.079
rno-miR-10a-5p	mature_unique	131	0.006	chr1_209040238_209040380	newMicroRNA_genome	1256	0.078
chr10_62782728_62782812	newMicroRNA_fasthomolog	129	0.006	chr3_18651980_18652124	newMicroRNA_genome	1249	0.078
hsa-miR-1303#ptr-miR-1303	maturehomolog_unique	128	0.006	rno-miR-181b-5p	mature_unique	1183	0.073
chr1_219153600_219153686	newMicroRNA_genome	127	0.006	chr10_67078689_67078839	newMicroRNA_homolog	1163	0.072
chrX_35821301_35821427	newMicroRNA_genome	123	0.006	chr10_67078715_67078809	newMicroRNA_fasthomolog	1158	0.072
rno-miR-652-3p	mature_unique	122	0.006	rno-miR-3557-5p	mature_unique	1156	0.072
chr8_113614814_113614902	newMicroRNA_fastgenome	112	0.005	rno-miR-423-3p	mature_unique	1150	0.071
chr8_113614807_113614909	newMicroRNA_genome	112	0.005	rno-miR-127-3p	mature_unique	1133	0.070
chr3_118996605_118996685	newMicroRNA_fasthomolog	112	0.005	rno-miR-23b-3p	mature_unique	1052	0.065
rno-miR-101a-3p	mature_unique	102	0.005	rno-let-7e-5p	mature_unique	1020	0.063
rno-miR-484	mature_unique	97	0.005	chr5_172899003_172899082	newMicroRNA_fastgenome	1019	0.063

These microRNAs showed more than 100 and ~1000 reads in blood and lung of the control rats respectively. Read count and percentage of the reads of individual microRNA in the sum of top 113 are also provided.

**Table 3:** A comparison of the profile of the most abundant microRNA in blood and lung found in the current study compared to those of Spornraft *et al*. and Caruso *et al*.

Most highly expressed (top20) miRNA											
Current study						Previous studies 16,17					
rat blood	read counts (n = 6)	% top 20	rat lung	read counts (n = 6)	% top 20	bovine blood	rpm^16^ (n = 9)	% top 10	male rat lung	absolute levels^17^(n = 5)	% top 20
rno-miR-486-5p	1940840	94.91	rno-miR-143-3p	174207	28.57	bta-miR-451	74917	27.5	rno-miR-26a	11526	10.1
rno-miR-92a-3p	23299	1.14	rno-miR-10a-5p	84796	13.91	bta-miR-486	58400	21.4	rno-miR-126	11056	9.7
rno-miR-191a-5p	21565	1.05	rno-miR-26a-5p	36273	5.95	bta-miR-25	40788	15	rno-let-7a	9747	8.5
rno-miR-16-5p	9957	0.49	rno-miR-30a-5p	35274	5.79	bta-miR-92a	38089	14	rno-let-7c	8762	7.7
rno-miR-3596a	7150	0.35	rno-miR-3596a	32088	5.26	bta-miR-191	13807	5.1	rno-let-7f	8290	7.2
rno-miR-25-3p	6881	0.34	rno-miR-99b-5p	28132	4.61	bta-let-7i	11134	4.1	rno-let-7b	7268	6.4
rno-miR-26a-5p	6610	0.32	rno-miR-486-5p	27940	4.58	bta-miR-185	9136	3.4	rno-miR-23b	6669	5.8
rno-miR-451-5p	4558	0.22	rno-let-7f-5p	24028	3.94	bta-miR-339a	8965	3.3	rno-miR-23a	6618	5.8
rno-let-7i-5p	3874	0.19	rno-miR-126a-5p	22723	3.73	bta-miR-26a	8823	3.2	rno-let-7d	6525	5.7
rno-miR-181a-5p	3794	0.19	rno-miR-181a-5p	20558	3.37	bta-miR-21-5p	8364	3.1	rno-miR-145	4950	4.3
rno-let-7f-5p	3021	0.15	rno-miR-146b-5p	17794	2.92				rno-let-7i	4293	3.8
rno-let-7c-5p	2780	0.14	rno-miR-22-3p	16162	2.65				rno-miR-30c	4085	3.6
rno-miR-151-3p	2393	0.12	rno-miR-125a-5p	15567	2.55				rno-miR-16	4051	3.5
rno-miR-27b-3p	2224	0.11	rno-miR-30d-5p	13955	2.29				rno-miR-30b-5p	3716	3.2
rno-let-7a-5p	1291	0.06	rno-miR-191a-5p	12568	2.06				rno-miR-125b-5p	3117	2.7
rno-let-7b-5p	1112	0.05	rno-miR-126a-3p	11732	1.92				rno-miR-195	3062	2.7
rno-miR-30a-5p	1041	0.05	rno-miR-27b-3p	10674	1.75				rno-let-7e	2978	2.6
rno-miR-140-3p	876	0.04	rno-miR-92a-3p	9513	1.56				rno-miR-24	2724	2.4
rno-miR-186-5p	812	0.04	rno-miR-151-3p	8838	1.45				rno-miR-26b	2709	2.4
rno-let-7d-5p	809	0.04	rno-let-7i-5p	6882	1.13				rno-miR-29a	2271	2.0

In the study of Spornraft *et al*.^16^, read counts were presented in reads per million (rpm) and n = 9; in the study of Caruso *et al*.^17^, counts were presented as absolute levels and n = 5; in the current study, total read counts were shown and n is 6 libraries, each of which included pooled RNA from 3 rats.

**Table 4:** Number of microRNA changed after radiation.

# of miRNA changed after radiation (p < 0.05)									
		↑				↓			
		1w	2w	3w	4w	1w	2w	3w	4w
Lung	1w	**62**	11	5		**40**	10	11	1
	2w	11	**24**	1	1	10	**16**	6	1
	3w	5	1	**22**	2	11	6	**64**	5
	4w		1	2	**4**	1	1	5	**8**
Blood	1w	**2**	1			**3**	1	1	1
	2w	1	**13**			1	**3**	1	1
	3w			**5**	3	1	1	**15**	7
	4w			3	**5**	1	1	7	**10**

↑ : increase after radiation; ↓ : decrease after radiation. w: week. p < 0.05 radiated vs. non-irradiated control. Egs: 62 microRNAs were found to be increased 1week after radiation in lung; 11 of the 62 were also increased at 2 weeks after radiation in lung, 5 of the 62 were also increased 3 weeks after radiation in lung. 24 microRNAs were found to be increased in lung at 2 weeks after radiation, of them 11 was also increased at 1week, as mentioned above, and 1 microRNA was also increased at 3 weeks, and 1 also increased at 4 weeks.
